# Room-temperature electrical control of polarization and emission angle in a cavity-integrated 2D pulsed LED

**DOI:** 10.1038/s41467-022-32292-2

**Published:** 2022-08-19

**Authors:** Juan Francisco Gonzalez Marin, Dmitrii Unuchek, Zhe Sun, Cheol Yeon Cheon, Fedele Tagarelli, Kenji Watanabe, Takashi Taniguchi, Andras Kis

**Affiliations:** 1 Institute of Electrical and Microengineering, École Polytechnique Fédérale de Lausanne (EPFL), CH-, 1015 Lausanne, Switzerland; 2 Institute of Materials Science and Engineering, École Polytechnique Fédérale de Lausanne (EPFL), CH-, 1015 Lausanne, Switzerland; 3 Research Center for Functional Materials, National Institute for Materials Science, 1-1 Namiki, Tsukuba, 305-0044 Japan; 4 International Center for Materials Nanoarchitectonics, National Institute for Materials Science, 1-1 Namiki, Tsukuba, 305-0044 Japan

**Keywords:** Lasers, LEDs and light sources, Two-dimensional materials

## Abstract

Devices based on two-dimensional (2D) semiconductors hold promise for the realization of compact and versatile on-chip interconnects between electrical and optical signals. Although light emitting diodes (LEDs) are fundamental building blocks for integrated photonics, the fabrication of light sources made of bulk materials on complementary metal-oxide-semiconductor (CMOS) circuits is challenging. While LEDs based on van der Waals heterostructures have been realized, the control of the emission properties necessary for information processing remains limited. Here, we show room-temperature electrical control of the location, directionality and polarization of light emitted from a 2D LED operating at MHz frequencies. We integrate the LED in a planar cavity to couple the polariton emission angle and polarization to the in-plane exciton momentum, controlled by a lateral voltage. These findings demonstrate the potential of TMDCs as fast, compact and tunable light sources, promising for the realization of electrically driven polariton lasers.

## Abstract (web summary)

2D semiconductors offer a promising platform for the realization of compact and CMOS-compatible optoelectronic components. Here, the authors report the realization of light-emitting diodes based on 2D WSe2 integrated with a planar cavity, showing the electrical control of the emission angle and polarization at room temperature.

## Introduction

Two-dimensional direct bandgap semiconductors are promising materials for on-chip integrated optoelectronic devices^1^. Since the demonstration of the first ultrasensitive photodetectors^2^ and light emitting diodes^3,4^, there has been a strong effort to improve the quantum efficiency, speed, energy consumption and wavelength range of 2D-based devices^5,6^. In recent years, integration with CMOS-compatible photonic platforms has proven the viability of the technology for the development of optical interconnects^7–9^. Furthermore, the integration of 2D materials with photonic cavities has enabled the demonstration of lasers^10,11^ and deterministic single photon emitters^12^, main building blocks for quantum information processing.

The spin-valley properties of TMDCs have also been exploited as a new degree of freedom, with the purpose of overcoming some of the fundamental limits related to Joule heating, speed and coherence in charge-based devices. The K and K’ valleys in monolayer TMDCs are coupled to circularly polarized light via the optical selection rules^13^, enabling the realization of polarization-sensitive photodetectors^14^ and light-emitting diodes^15,16^. However, so far, these devices required cryogenic temperatures and complex fabrication.

Although practical applications of LEDs for information processing require fast electrical switching and low energy consumption^17^, most devices based on 2D materials operate with direct current (DC) and present very low values of radiant efficiency, due to the large contact resistance and low quantum efficiency. The control of the emission pattern is also fundamental for the coupling to photonic waveguides and detectors, as well as for illumination applications^18^. Yet, the location and directionality of light emitted from TMDCs could only be controlled by the coupling with photonic cavities and meta-surfaces, where the far field emission pattern is fixed by the geometry of the structure^19^.

Here, we overcome these limitations by integrating a pulsed light emitting diode (LED) based on monolayer WSe_2_ in a distributed Bragg reflector (DBR) cavity. By applying a square train of pulses to a source electrode^20,21^, we show MHz operation with the emission of ns pulses. In addition, we demonstrate electrical control of the emission angle for valley-polarized photons at room temperature. Together, our results establish a method to electrically modulate the location, directionality and polarization of 2D LEDs, with switching times ultimately limited by the pulse linewidth.

## Results

### Device design and basic characterization

**Fig. 1:**
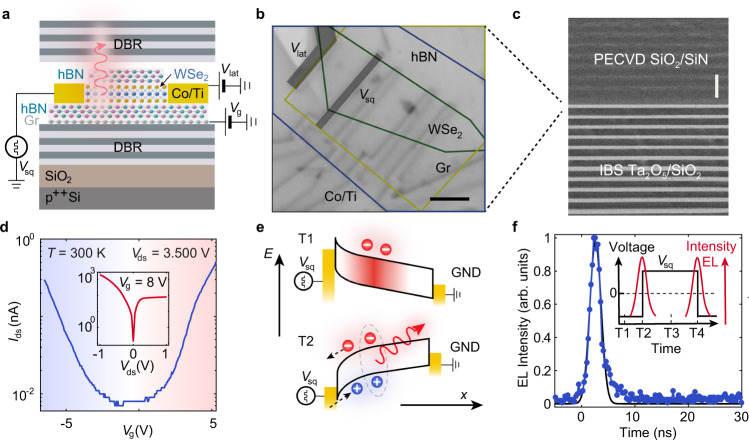
Device structure and basic characterization. **a** Schematics of the device structure. *V*_sq_: square voltage pulse. *V*_lat_: lateral voltage. *V*_g_: gate voltage. **b** Optical image of the completed device. Scale bar, 4 µm. **c** Scanning electron microscopy (SEM) image of the distributed Bragg reflector (DBR) cavity. Scale bar, 500 nm. **d** Electrical transport properties of the device, showing ambipolar behavior. Inset shows the drain-source current *I*_ds_ as a function of the drain-source bias voltage *V*_ds_ **e** Schematics of excitonic emission driven by a high frequency square voltage pulse. Red (blue) circles correspond to electrons (holes). The red arrow indicates light emission after exciton (dashed line) recombination. The black arrows correspond to the position (*x*) and energy (*E*) axis. **f** Time-resolved measurement of electroluminescence (EL) intensity for a square voltage with peak-to-peak amplitude ${V}_{{{{{{\rm{sq}}}}}}}^{{{{{{\rm{pp}}}}}}}=15.5\,{{{{{\rm{V}}}}}}$ and frequency *f* = 8 MHz. Inset shows the correlation between EL emission and voltage applied to the contact.

Our device consists of a WSe_2_ monolayer encapsulated with a thick bottom h-BN and top trilayer h-BN. Monolayer graphene is used as a transparent gate electrode and top Co/Ti contacts are employed for electrical injection through the thin h-BN tunneling barrier (Fig. 1a). Figure 1b shows the optical image of the device after contact deposition (See Methods). The uniform and high-quality factor *λ*/2 cavity (Supplementary Figs. 1, 2) is formed by a bottom mirror consisting of 12 pairs of Ta_2_O_5_/SiO_2_ and a top mirror consisting of 9 pairs of SiO_2_/SiN (Fig. 1c). The optical properties of the mirrors are analyzed in detail in Supplementary Fig. 3. The cavity structure is designed such that monolayer WSe_2_ is located at the maximum of the electric field (Supplementary Fig. 4).

Figure 1d demonstrates electron and hole transport for the cavity integrated WSe_2_ at room temperature (RT). The efficient electrostatic gating is confirmed by the gate-dependent PL intensity (Supplementary Figs. 5, 6). To achieve electrically driven light emission, we apply a high frequency square pulse (*V*_sq_) with rise time *τ*_rise_ < 10 ns to the source contact while we ground both the drain and the gate. The mechanism behind pulsed electroluminescence (EL) is depicted in Fig. 1e. At time T1 (Fig. 1f, inset), the monolayer is initially n-doped. When *V*_sq_ switches to positive values with a rising time on the order of ns, the chemical potential of the monolayer cannot react as fast as the Fermi level of the metal, leading to a steep band bending at the interface^21^. Consequently, an electron tunneling current flows towards the contact while a hole tunneling current flows outwards from it. The spatial overlap between electron and hole wavefunctions at T2 leads to the formation of excitons and subsequent light emission. This mechanism produces the emission of short pulses with frequencies in the MHz range and duration below *τ*_FWHM_ = 3 ns, as shown in Fig. 1f. The maximum operation speed can therefore reach $1/{\tau }_{{{{{{\rm{FWHM}}}}}}}=0.\dot{3}\,{{{{{\rm{GHz}}}}}}$, limited by the device geometry and rising time of the pulse generator.

### Exciton-photon coupling

**Fig. 2:**
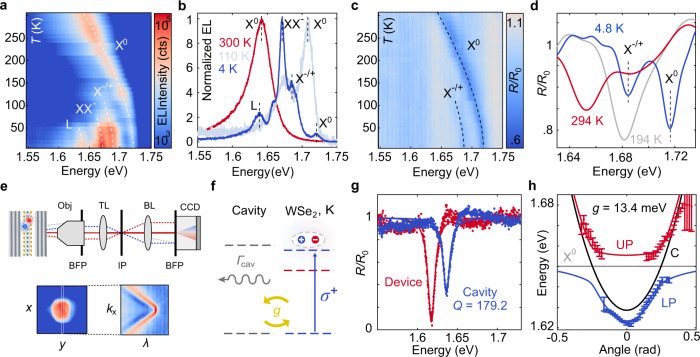
Excitonic light emission and strong coupling regime. **a**, **b** Temperature dependence of EL at ${V}_{{{{{{\rm{sq}}}}}}}^{{{{{{\rm{pp}}}}}}}=13.5\,{{{{{\rm{V}}}}}}$ and *f*  = 9 MHz. X^0^: neutral exciton, X^−/+^: charged exciton. XX^−^: negatively charged biexciton. L: bound exciton. Dashed lines are guides to the eye indicating the different exciton energies. **c**, **d** Temperature dependence of reflectance before cavity growth. R_0_: background reflectance taken on the heterostructure without WSe_2_. **e** Schematics of the setup used for back focal plane spectroscopy. BFP back focal plane, TL tube lens, IP image plane, BL Bertrand lens. The surface plots correspond to the reflected light intensity measured at the image plane and back focal plane. **f** Physical model for exciton–photon coupling. A cavity mode with decay rate *Γ*_cav_ interacts with coupling strength **g** with an exciton in the K valley, generated by a circularly polarized (*σ*^+^) laser. **g** Reflectance measured on the bare cavity (DBR/Gr/hBN/hBN/DBR) and device (DBR/Gr/hBN/WSe_2_/hBN/DBR). Continuous lines represent Lorentzian fits to the data, from where we extract the linewidth *γ*_c_ = 9.132 ± 0.007 meV and quality factor *Q* = 179.2 ± 0.5 of the cavity mode. **h** Energy dispersion on the heterostructure with WSe_2_ together with fits to the data using a coupled oscillator model. The energies and standard errors are extracted from Lorentzian fits of the reflectance spectra. The upper (red, UP) and lower (blue, LP) polariton branches are clearly visible. The black and gray lines correspond to the energy dispersion of the bare exciton and cavity modes.

Before top cavity growth, the high-quality hBN encapsulation allows us to achieve narrow excitonic emission at low temperature (Fig. 2a). At *T* = 5 K, we identify neutral exciton (X^0^), charged exciton (X^−/+^), negatively charged biexciton (XX^−^) and bound exciton (L) emission^20^, whereas the neutral exciton dominates the spectrum at *T* = 300 K. Similar results are obtained in PL measurements (Supplementary Figs. 5, 6). Temperature also modifies the exciton oscillator strength and exciton lifetime, crucial for reaching the strong coupling regime^22,23^. In Fig. 2c, we plot the temperature dependence of the normalized reflectance at *V*_g_ = 0 V. There is a clear correlation between reflectance and EL, with neutral and charged exciton absorption peaks appearing at low temperatures. The oscillator strength is proportional to the reflection contrast and linewidth^24^, and remains high at room temperature.

The top DBR growth results in a uniform cavity mode at the device^25^ (Supplementary Fig. 7). For the bare cavity with hBN, the quality factor is *Q* = 296.9 ± 0.5 (Supplementary Fig. 8). The addition of the monolayer graphene gate reduces the quality factor to *Q* = 179.2 ± 0.5, with a cavity linewidth *γ*_c_ = 9.132 ± 0.006 meV (Fig. 2g). To quantify the coupling between WSe_2_ excitons and the cavity mode, the setup is modified to image the back focal plane of the objective^26^, as indicated in Fig. 2e. The energy dispersion of the heterostructure consisting of DBR/Gr/hBN/WSe_2_/hBN/DBR is plotted in Fig. 2h. The data points are obtained from Lorentzian fits to the reflectance dispersion (Supplementary Fig. 8). There is a clear Rabi splitting with the formation of a lower (LP) and upper (UP) polariton branches, coming from the strong coupling between the cavity mode and WSe_2_ excitons. Numerical simulations considering a coupled oscillator model^27^ (see Methods) allow us to reproduce the experimental results and extract a coupling strength *g* = 13.4 ± 1.0 meV, with a detuning between the cavity mode and the exciton energy *Δ* = *E*_c_ − *E*_X_ = −21 meV. The proximity to metal contacts and the presence of the graphene gate reduces the coupling strength compared to previous reports^28,29^. Supplementary Fig. 11 shows further proof of the strong coupling by measuring the Zeeman splitting under external magnetic fields.

### Electrical control of light emission

**Fig. 3:**
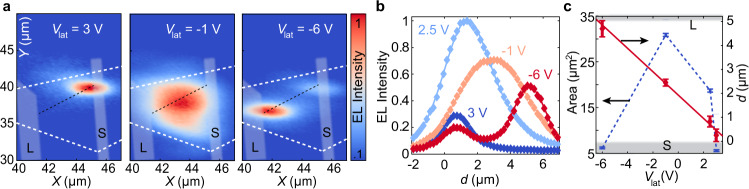
Electrical control of the spatial emission profile. **a** Spatial EL emission pattern integrated over the whole spectral range at different lateral voltages *V*_lat_. The source (S) and lateral (L) contacts are highlighted in gray. White dashed lines indicate the WSe_2_ edges. **b** Normalized EL intensity as a function of the distance from the source electrode (*d*), following the black dashed line in panel (**a**). **c** EL emission area at 1/e intensity and distance from the source contact, extracted from Gaussian fits to the data in panel (**b**). Error bars represent the standard error. The gray rectangles indicate the position of the contacts.

After characterizing the optical and electrical properties of the LED, we further apply a lateral voltage on the monolayer WSe_2_ to electrically control the spatial, angular and polarization properties of emitted light at room temperature. With the application of a finite voltage on a second contact deposited on the flake, the band alignment and the total external electric field are modified. Both parameters have a direct impact on the velocity and location of carriers before recombination, which enables the electrical displacement of the polariton cloud, as shown in Fig. 3. The spatial emission profile for three representative lateral voltages *V*_lat_ is shown in Fig. 3a. The cross section along the propagation direction is plotted in Fig. 3b, where *d* = 0 μm denotes the position of the source electrode. By performing a Gaussian fitting of the EL intensity profile, it is possible to extract the EL area and position, which is linearly dependent on *V*_lat_ (Fig. 3c). We note that the conformal growth of the top mirror ensures a high cavity *Q*-factor even when emission occurs close to the contact edge (See Supplementary Fig. 7). The uniformity of the cavity mode and coupling strength is evidenced by measuring the polariton emission energy along the propagation direction (Supplementary Fig. 10).

The lateral voltage also determines the resulting emission angle of photons out of the cavity, by modifying the momentum of electrons and holes leading to the formation of polaritons. Since the movement of carriers in WSe_2_ can be described by diffusive transport, their velocity is given by their mobility and the electric field: ${v}_{{{{{{\rm{e}}}}}},{{{{{\rm{h}}}}}}}= \pm \,{\mu }_{{{{{{\rm{e}}}}}},{{{{{\rm{h}}}}}}}E={\pm \mu }_{{{{{{\rm{e}}}}}},{{{{{\rm{h}}}}}}}{V}_{{{{{{\rm{lat}}}}}}}/w$, with *w* the distance between electrodes. Since the kinetic energy of single carriers is much lower than the exciton binding energy, excitons will be generated as long as electrons and holes spatially overlap. This remains valid despite the large lateral voltages applied to the sample, since the electric field always remains lower than the dissociation field for excitons E_max_ < E_diss_^30^. Considering that the scattering rate for electrons and holes is different, the exciton momentum is ${{{{{{\bf{p}}}}}}}_{{{{{{\rm{X}}}}}}}={m}_{{{{{{\rm{e}}}}}}}^{*}\,{{{{{{\bf{v}}}}}}}_{{{{{{\rm{e}}}}}}}+{m}_{{{{{{\rm{h}}}}}}}^{*}\,{{{{{{\bf{v}}}}}}}_{{{{{{\rm{h}}}}}}}\ne 0$, with m^*^ the carrier effective mass.

**Fig. 4:**
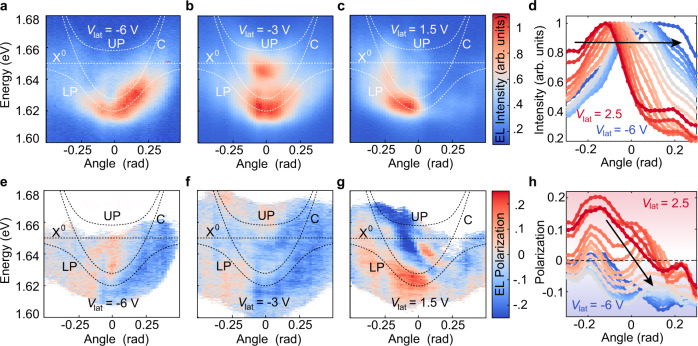
Electrical control of the emission angle and polarization. **a**–**c** EL dispersion as a function of emission angle at three different lateral voltages. **d** Emission intensity along the calculated LP branch displayed as a function of the emission angle. White dashed lines correspond to the energy dispersion of the upper and lower polariton (UP, LP), cavity mode (C) and neutral exciton (X^0^). **e**–**g** Polarization-resolved EL dispersion as a function of emission angle at three different lateral voltages. Data points with emission intensity comparable to the background counts are not shown. **h** Circular polarization along the calculated LP branch displayed as a function of the emission angle. The data shown in (**d**, **h**) is averaged over an energy *ΔE* = 1/2 *k*_B_ *T*.

Excitons with a finite in-plane momentum relax to the LP branch, where emission out of the cavity occurs through the photonic component, with ${{{{{{\rm{p}}}}}}}_{{{{{{\rm{LP}}}}}}}={{{{{{\rm{p}}}}}}}_{{{\gamma }}}={{\hslash }}{k}_{//}$. The in-plane momentum ${k}_{//}$ is related to the photon emission angle *θ* by ${k}_{//}=\frac{2{{\pi }}{n}_{{{{{{\rm{c}}}}}}}}{{\lambda }_{0}}{{\tan }}{\theta }_{{{{{{\rm{c}}}}}}}$, with *n*_c_ sin *θ*_c_ = sin *θ*, and *θ*_c_ the angle inside the cavity^31^. This implies that the polariton emission angle is linearly proportional to the applied lateral voltage to a first order approximation (Supplementary Fig. 12). The EL dispersion as a function of *θ* is plotted in Fig. 4a–c for three representative values of the applied lateral voltage. The lateral voltage ${V}_{{{{{{\rm{lat}}}}}}}^{0}=-\!3\,{{{{{\rm{V}}}}}}$ for which EL is emitted normal to the 2D plane of the monolayer is different from zero because the dynamics of carriers is also affected by the voltage *V*_sq_ applied to the source. When ${V}_{{{{{{\rm{lat}}}}}}} > {V}_{{{{{{\rm{lat}}}}}}}^{0}$, the electrically generated excitons acquire a net momentum in the −***x*** direction, and photons are emitted with an angle *θ* < 0 with the direction perpendicular to the monolayer plane. Conversely, ${V}_{{{{{{\rm{lat}}}}}}}\, < \,{V}_{{{{{{\rm{lat}}}}}}}^{0}$ leads to photons emitted with angles *θ* > 0. The energy and momentum conservation conditions are relaxed at room temperature (*k*_B_ *T* ~ 26 meV) due to phonon scattering^32^, which results in photons with a broad distribution of energy and momentum for a fixed value of the lateral voltage, as shown in Fig. 4.

Together with the photon emission angle, we can also electrically control the EL polarization at room temperature. Electrical control of EL polarization has so far only been demonstrated for electric double layer transistors based on WSe_2_. These devices, however, require an ionic liquid and can only be operated in DC at cryogenic temperatures^15^. Figure 4e–g shows the momentum and energy resolved polarization for EL at three representative values of the lateral voltage. The circular polarization can be tuned from *ρ*_EL_ > 20% to *ρ*_EL_ <−20%. The origin of the observed polarization comes from the anisotropic band dispersion of carriers in WSe_2_ (see Methods). Under the application of a lateral electric field, the electron and hole distributions shift in momentum space. This shift induces an energy splitting between K and K’ excitons, which is proportional to the in-plane momentum. Exciton relaxation to the lowest energy state in one of the valleys before scattering to the polariton can then explain the observed valley polarization (Supplementary Fig. 15). The direction of the electric field with respect to the crystal lattice is crucial for achieving a high degree of polarization. In our case, the contacts are deposited perpendicularly to the long straight edge of the WSe_2_ flake, which tends to have either armchair or zigzag edges^33^. Supplementary Fig. 13 shows the EL polarization as a function of the driving voltage amplitude *V*_sq_ before the cavity growth, which supports the trigonal warping effect^15,34^ as the origin of the polarization change in our device (relevant for fields on the order of 1–10 V/μm) with negligible contribution from the nonlinear valley and spin currents (with a quadratic electric field dependence). Further proof for the origin of the polarization is shown in Supplementary Fig. 14, where a second device is fabricated with contacts deposited along the armchair and zigzag directions of the monolayer. In this device, the polarization is only observable for the contact placed along the armchair direction, as expected from the trigonal warp effect.

### Device operation

**Fig. 5:**
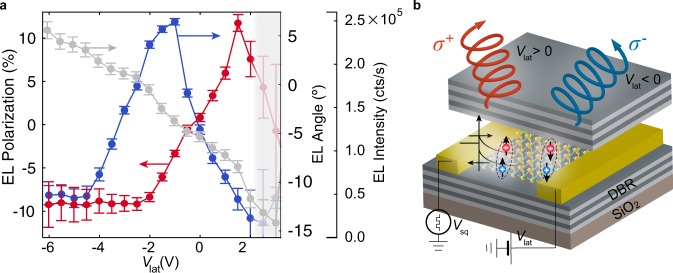
Device operation. **a** Weighted arithmetic mean of EL angle (gray), polarization (red) and integrated intensity (blue) extracted from the dispersion maps in Fig. 4. Error bars represent the standard error. The gray box covers the data points for which the detected photon counts are of the same order of magnitude as the noise. **b** Schematic representation of the device operation, where the properties of emitted light can be electrically controlled.

Figure 5a shows the weighted arithmetic mean of the polarization for each value of the lateral voltage (see Methods), which follows a linear behavior for moderate values of the lateral voltage. The quasi-resonant electrical injection (see Methods) together with a strong coupling to the cavity mode can explain the large degree of circular polarization shown in Fig. 5^35^. In most experiments, non-resonant optical excitations are used to create a population imbalance in the K and K’ valleys. However, these high-energy excitons have a large center-of-mass wave vector. Under these conditions, there is a fast depolarization due to the long-range Coulomb exchange interaction from electrons and holes^36^. Furthermore, the L-T energy splitting (for excitons with their dipole moment parallel and perpendicular to the wave vector) increases linearly with the wave vector, leading to a fast depolarization^37^. The lateral voltage, on the other hand, creates valley-polarized carriers with a relatively small energy and momentum with respect to the conduction and valence band edges. In addition, the large spatial extent of the polariton wavefunction^38^ reduces the intravalley scattering due to the disorder potential^39^, resulting in a longer valley pseudospin relaxation time compared to intralayer excitons^37,40^. Furthermore, the negative detuning in our cavity reduces the intervalley scattering, which only occurs through the excitonic component. To exclude the possibility that the observed polarization and directionality arises from the displacement of EL with respect to the focal point of the objective, we perform a calibration experiment (Supplementary Fig. 16), discussed in Methods. We further demonstrate that the spatial variability of the cavity coupled exciton emission angle and polarization is negligible compared to the modulation achieved by the lateral voltage (Supplementary Fig. 17).

## Discussion

The successful integration of electrically tunable light emitting diodes in planar cavities achieved in this work (Fig. 5b) constitutes a crucial step in the development of practical devices to study light-matter interaction, where the frequency, position, directionality, and polarization can be tuned electrically at room temperature. This tunability would facilitate the coupling of EL to other photonic structures such as diffraction gratings, waveguides and resonators, and can relax the alignment challenges of optical to electrical interconnects. The simplicity of the method for EL generation implies that large-area CVD grown TMDCs with metal contact arrays and top cavity growth could be used to achieve high integration densities. Furthermore, the large tunneling current per voltage pulse together with the high frequency operation could lead to a transient population inversion and electrically driven lasing. In the regime of strong-coupling, polariton lasing could be achieved at lower thresholds, with an electrically tunable chirality^41^.

## Methods

### Device fabrication

The substrate consists of a DBR mirror formed by 12 pairs of Ta_2_O_5_/SiO_2_ layers deposited by ion beam sputtering (IBS) on top of a doped silicon wafer. Single layer graphene flakes for the bottom gate were obtained by mechanical exfoliation from graphite (NGS) on the DBR substrates and patterned to the final shape by electron beam lithography and oxygen plasma etching. Few-layer h-BN and monolayer WSe_2_ (HQ Graphene) were stacked using the dry polymer-assisted transfer^42^ after exfoliation on a viscoelastic stamp. The thin h-BN encapsulation and tunneling layer was transferred using a wet polymer-assisted transfer after exfoliation on a polymer double layer^43^. The thickness of the bottom and top hBN is 29 nm and 1 nm, respectively. After the stack was completed, the heterostructure was thermally annealed at 340 °C under high vacuum (10^−6^ mbar) for 12 h. Co (35 nm)/Ti (10 nm) contacts were defined perpendicularly to one of the WSe_2_ crystal axes by electron-beam lithography and deposited by electron beam evaporation. The device was again annealed at 250 °C under high vacuum (10^−6^ mbar) for 12 h. Finally, the top SiO_2_ spacer and nine pairs of SiO_2_/Si_3_N_4_ layers were deposited by plasma enhanced chemical vapor deposition (PECVD). The second device analyzed in Supplementary Fig. 11 was fabricated following the same steps as the main device, but Ti (2 nm)/Au (80 nm) metal contacts were directly deposited on top of WSe_2_ before the transfer of the hBN encapsulation layer.

### Optical and electrical measurements

All measurements presented in this work were performed under vacuum at a temperature of 300 K, unless specified otherwise. Reflectance measurements were taken by focusing a fiber- coupled white light source onto a 2.5 µm spot on the sample. The incident power was 90 µW. Photoluminescence was generated by excitation with a continuous-wave 647 nm laser diode focused to a diffraction-limited spot size of about 1 µm. The incident power was 200 µW. The spectral and spatial characteristics of the device emission were analysed simultaneously. The emitted light was acquired using a spectrometer (Andor Shamrock with Andor Newton CCD camera), and the laser line was removed with a long-pass 650 nm edge filter. For spatial imaging, light was collected using an Andor Ixon CCD camera after passing through a long-pass 700 nm edge filter. For polarization-resolved measurements, a rotator with a *λ*/2 plate was used to control the polarization incident on a *λ*/4 plate between the sample and the objective. A calcite beam displacer was placed before the spectrometer to separate the two measured polarizations. Angular resolved measurements were obtained by imaging the Fourier plane on the CCD camera after the spectrometer. Two convergent lenses are used to image the back focal plane of the objective, with the spectrometer slit closed to 10 µm. Electroluminescence was generated by applying a square voltage wave using an Agilent 33520 function generator. Time-resolved EL measurements were obtained with a silicon avalanche photodiode (APD) from Excelitas (SPCM-AQRH) and time correlated single photon counting (TCSPC) system (Picoharp 300).

### Cavity reflectance and exciton-photon coupling

The normalized reflectance for the cavity mode is plotted in Fig. 2g. The back reflected intensity measured when exciting from the top mirror (*R*_t_) follows the Airy distribution^44^:

1 $$\begin{array}{c}\frac{{I}_{{{{{{\rm{ref}}}}}}}}{{I}_{{{{{{\rm{inc}}}}}}}}=\frac{{\left(\sqrt{{R}_{{{{{{\rm{t}}}}}}}}-\sqrt{{R}_{{{{{{\rm{b}}}}}}}}\right)}^{2}+4\sqrt{{R}_{{{{{{\rm{t}}}}}}}{R}_{{{{{{\rm{b}}}}}}}}{{{\sin }}}^{2}\phi }{{\left(1-\sqrt{{{R}_{{{{{{\rm{t}}}}}}}R}_{{{{{{\rm{b}}}}}}}}\right)}^{2}+4\sqrt{{R}_{{{{{{\rm{t}}}}}}}{R}_{{{{{{\rm{b}}}}}}}}{{{\sin }}}^{2}\phi }\end{array}$$

With 2*ϕ* = 2π*νt*_RT_, *ν* the frequency and *t*_RT_ the round-trip time in the cavity. We use this equation to fit the reflectance spectra and extract the linewidth and resonant energy. The reflectance dispersion for the bare cavity and device heterostructure is plotted in Supplementary Fig. 8. The cavity mode energy is calculated as ${E}_{{{{{{\rm{c}}}}}}}={E}_{{{{{{\rm{ph}}}}}}}/\sqrt{1-{\left(sin\,\theta/n_c\right)}^{2}}$, with *E*_ph_ the photon energy and ${n}_{{{{{{\rm{c}}}}}}}=\frac{{n}_{{{{{{\rm{SiO}}}}}}2} {t}_{{{{{{\rm{SiO}}}}}}2}+{n}_{{{{{{\rm{hBN}}}}}}} {t}_{{{{{{\rm{hBN}}}}}}}}{{t}_{{{{{{\rm{SiO}}}}}}2}+{t}_{{{{{{\rm{hBN}}}}}}}}=1.538$ the effective refractive index based on the filling fractions of SiO_2_ and hBN^45^.

The reflectance dispersion in Supplementary Fig. 8 is fitted with a double Lorentzian function to extract the energies of the polariton branches. The energy dispersion is then fitted according to equation^27^:

2 $$\begin{array}{c}{E}_{{{{{{\rm{UP}}}}}},{{{{{\rm{LP}}}}}}}=\frac{1}{2}\left({E}_{{{{{{\rm{X}}}}}}}+{E}_{{{{{{\rm{C}}}}}}}-i\left({\gamma }_{{{{{{\rm{c}}}}}}}+{\gamma }_{{{{{{\rm{X}}}}}}}\right)\right)\pm \frac{1}{2}\sqrt{4{g}^{2}+{\left({E}_{{{{{{\rm{X}}}}}}}-{E}_{{{{{{\rm{c}}}}}}}+i\left({\gamma }_{{{{{{\rm{c}}}}}}}-{\gamma }_{{{{{{\rm{X}}}}}}}\right)\right)}^{2}}\end{array}$$

For the fitting, the exciton and cavity linewidths are fixed, and take values *γ*_c_ = 9.132 ± 0.007 meV (Lorentzian fits from Fig. 2g) and *γ*_X_ = 27.9 ± 0.2 meV (Gaussian fits from Supplementary Fig. 6). The coupling strength extracted from the fitting is *g* = 13.4 ± 1.0 meV. The Rabi splitting can then be calculated as ℏ*Ω* = 19 ± 3 meV, with $\hslash \varOmega=\hslash \sqrt{4{g}^{2}-{\left({\gamma }_{{{{{{\rm{c}}}}}}}-{\gamma }_{{{{{{\rm{X}}}}}}}\right)}^{2}}$. The condition for strong coupling $g \, > \, \frac{\left|{\gamma }_{{{{{{\rm{X}}}}}}}-{\gamma }_{{{{{{\rm{c}}}}}}}\right|}{2}=9.3\pm 0.1\,{{{{{\rm{meV}}}}}}$ is therefore satisfied in our sample. In addition, for the two resonances to be spectrally separable, the energy splitting needs to be larger than the sum of the half-linewidths: ℏ*Ω* > (*γ*_X_ + *γ*_c_)/2 = 18.5 ± 0.1 meV. The fact that the Rabi splitting is comparable to the half-linewidths makes the observation of strong coupling difficult in our sample.

The measured splitting in reflectance is usually smaller than the intrinsic energy splitting of polariton states. In the high reflectivity limit, and assuming exciton-cavity resonance^27^:

3 $$\begin{array}{c}\hslash {\varOmega }_{{{{{{\rm{ref}}}}}}}=2\sqrt{\sqrt{{g}^{4}{\left(1+\frac{2{\gamma }_{{{{{{\rm{X}}}}}}}}{{\gamma }_{{{{{{\rm{c}}}}}}}}\right)}^{2}+2{g}^{2}{\gamma }_{{{{{{\rm{X}}}}}}}^{2}\left(1+\frac{{\gamma }_{{{{{{\rm{X}}}}}}}}{{\gamma }_{{{{{{\rm{c}}}}}}}}\right)}-\frac{2{g}^{2}{\gamma }_{{{{{{\rm{X}}}}}}}}{{\gamma }_{{{{{{\rm{c}}}}}}}}-{\gamma }_{{{{{{\rm{X}}}}}}}^{2}}\end{array}$$

This equation can be used to recognize the underestimation of *g* in reflectance measurements. Considering the observed Rabi splitting of ℏ*Ω*_ref_ = 19 ± 3 meV and the experimental values of *γ*_X_ and *γ*_c_, the coupling strength can be derived from the above equation as *g*_i_ = 21.3 ± 0.6 meV. The coupling strength best describing the EL energy dispersion in Fig. 4 is *g*_EL_ = 17.4 ± 0.9 meV = (*g*_ref_ + *g*_i_)/2.

### Pulsed electrical injection

The transient origin of current injection results in a localized emission close to the source electrode. However, as shown in Fig. 3a, the intensity maximum is not centered at the contact, due to strong lateral electric fields that deplete the electron and hole populations away from the electrode. The initial tunneling of electrons and holes can generate hot carriers with a large wave-vector and energy above the bandgap. On the other hand, electrons and holes recombine at a distance *d* > 500 nm from the contact (Fig. 3a). This implies that carriers need a travel time *τ*_t_ = *d*·*w*/(*μ*_e,h_·*V*_lat_) > 5 ps to reach the recombination spot, with *μ*_e,h_ taken from^46^. Therefore they relax from the excited injection condition before exciton formation occurs (scattering rate *τ*_s_ ≪ *τ*_t_)^47^.

### Valley polarization of carriers under external electric fields

Lateral electric fields shift the distribution of electrons and holes in *k*-space, with a distribution function for carriers that can be phenomenologically described by^15^:

4 $$\begin{array}{c}{f}_{{{{{{\rm{c}}}}}},{{{{{\rm{v}}}}}}}\left({{{{{\bf{k}}}}}}\right)={f}_{{{{{{\rm{c}}}}}},{{{{{\rm{v}}}}}}}^{0}\left({{{{{\bf{k}}}}}}-\frac{q\tau }{{{\hslash }}}{{{{{\bf{E}}}}}}\right)\end{array}$$

where ${f}_{{{{{{\rm{c}}}}}},{{{{{\rm{v}}}}}}}^{0}\left({{{{{\bf{r}}}}}},{{{{{\bf{k}}}}}}\right)={N}_{{{{{{\rm{c}}}}}},{{{{{\rm{v}}}}}}}{{\exp }}\left(-\frac{|{\epsilon }_{{{{{{\rm{c}}}}}},{{{{{\rm{v}}}}}}}\left({{{{{\bf{k}}}}}}\right)-{\mu }^{{{{{{\rm{c}}}}}},{{{{{\rm{v}}}}}}}\left({{{{{\bf{r}}}}}}\right){{\vert }}}{{k}_{{{{{{\rm{B}}}}}}}{T}_{{{{{{\rm{eff}}}}}}}}\right)$ is the distribution of electrons and holes at the band extrema. *N*_c,v_ is the normalization factor, *ϵ*_c,v_ (**k**) the band dispersion, *μ*^c,v^ (**r**) the chemical potential and *T*_eff_ the effective carrier temperature. The relaxation time is given by $\tau=\frac{{\mu }_{{{{{{\rm{c}}}}}},{{{{{\rm{v}}}}}}}{m}_{{{{{{\rm{c}}}}}},{{{{{\rm{v}}}}}}}^{*}}{\left|q\right|}$, with *μ*_c,v_ the carrier mobility and ${m}_{{{{{{\rm{c}}}}}},{{{{{\rm{v}}}}}}}^{*}$ the carrier effective mass. 

The energy dependent emission intensity resulting from electron-hole recombination is given by the Fermi golden rule, which for a constant dipole moment takes the form:

5 $$\begin{array}{c}I\left(E \right)\propto \int {{\delta }}{\left[{\left(E -{\epsilon }^{{{{{{\rm{c}}}}}}}\left({{{{{\bf{k}}}}}}\right)+{\epsilon }^{{{{{{\rm{v}}}}}}}\left({{{{{\bf{k}}}}}}\right)\right)}^{2}+{\delta }^{2}\right]}^{-1}{f}_{{{{{{\rm{v}}}}}}}\left({{{{{\bf{k}}}}}}\right){f}_{{{{{{\rm{c}}}}}}}\left({{{{{\bf{k}}}}}}\right){{{{{{\rm{d}}}}}}}^{2}{{{{{\bf{k}}}}}}\end{array}$$

where *δ* represents the broadening. Due to the anisotropy in the valence band of TMDCs at the K and K’ valleys, the resulting luminescence is circularly polarized: ${I}_{{{{{{\rm{K}}}}}}}\left(E \right)\;\ne\; {I}_{{{{{{\rm{K}}}}}}{{\hbox{'}}}}\left(E \right).$ This mechanism is responsible for the valley polarization observed in the light emitting diode without the cavity (Supplementary Figs. 13, 14). However, this model is not valid in the strong coupling regime. The exciton-polariton valley polarization requires a more complex kinetic equation considering relaxation rates from the exciton reservoir to the polariton states, exciton-cavity detuning, coherence and valley lifetimes. A theoretical model for the case of TMDCs integrated in optical cavities has been previously developed^38^. This model can be combined with the anisotropic band dispersion of carriers to understand the observed valley polarization. In Supplementary Fig. 15 we show a simplified model in one dimension in reciprocal space to explain the effect of a lateral electric field on the exciton dispersion and polariton polarization. First, we plot the energy dispersion for single carriers at the K and K’ valleys taking into account the trigonal warp effect by considering a two-band k·p model up to third order in the crystal momentum^48,49^. For simplicity, we focus on the crystal direction *k*_*x*_, with *x* being the axis along the zigzag direction of the lattice, where the electric field is applied. The exciton dispersion for both valleys at zero electric field is then simply calculated from the electron and hole energies. Even at zero field, there is a sizable splitting at large in-plane momentum. However, both K and K’ excitons are degenerate in energy at the bottom of the bands. When applying an in-plane electric field, the Fermi levels of electrons and holes shift in momentum space by $\frac{q\tau }{\hslash }E$, as described before. For an applied field of *E* = −1 V/μm and relaxation rate^50^ *τ* = 150 fs, the exciton dispersion becomes highly anisotropic for the K and K’ valleys, as shown in Supplementary Fig. 15c. Excitons generated at *k*_*x*_ > 0 then relax to the K’ valley, which becomes the lowest energy state. These parameters represent the experimental conditions in Fig. 4a, e. The magnitude of this effect is strongly dependent on the carrier relaxation rate. Taking *τ* = 400 fs, the exciton energy splitting between the K and K’ valleys can reach *ΔE* = 5 meV. This simple model can qualitatively explain the observed valley polarization, coming from exciton relaxation to one of the valleys before scattering to the polariton state.

### Simulations of exciton–photon momentum and energy conservation

To theoretically compute the photon emission angle as a function of the lateral electric field, we apply momentum conservation to the recombination process (${{p}_{{{\gamma }}}=p}_{{{{{{\rm{LP}}}}}}}={p}_{{{{{{{\rm{X}}}}}}}^{0}}$). Here we neglect Coulomb interactions and phonon scattering. The photon momentum can be calculated from its emission angle with respect to the normal direction to the monolayer plane *θ*, as described in the main text. The exciton momentum is calculated from the electron and hole velocities, ${p}_{{{{{{{\rm{X}}}}}}}^{0}}={m}_{{{{{{\rm{e}}}}}}}^{*} {v}_{{{{{{\rm{e}}}}}}}+{m}_{{{{{{\rm{h}}}}}}}^{*} {v}_{{{{{{\rm{h}}}}}}}$. Carrier velocities are limited by the mobility in the diffusive regime, with *v*_e,h_ = *μ*_e,h_*V*_lat_/*w*, where *μ*_e,h_ is the carrier mobility, *V*_lat_ the lateral voltage and *w* is the width of the channel. The effective mass for electrons and holes is taken as ${m}_{{{{{{\rm{h}}}}}}}^{*}=0.51\,{m}_{{{{{{\rm{e}}}}}}}$ and ${m}_{{{{{{\rm{e}}}}}}}^{*}=0.39\,{m}_{{{{{{\rm{e}}}}}}}$^47^. The dependence of the photon emission angle with the lateral field is calculated in Supplementary Fig. 12 for different values of the electron and hole mobilities. It should be noted that the simulations can only qualitatively describe the observed change in the photon emission angle. A more detailed description considering the carrier dynamics at the metal–semiconductor junction would be needed for a quantitative description but is outside of the scope of this work.

### Reference experiment

To prove that the observed polarization and directionality is not an artifact related to the spatial displacement of EL at different lateral voltages, we perform an experiment where the objective position is scanned for fixed EL emission conditions. Supplementary Fig. 16 demonstrates that the movement of the focal point with respect to the EL location cannot explain the large change in emission angle and polarization observed in the main text.

### Magnetic field dependence of electroluminescence

Further proof for the presence of strong coupling in our device is given by the external response of the LP state to an external magnetic field, which is shown in Supplementary Fig. 11. The exciton and photon fractions in the LP state are given by the Hopfield coefficients^51^, which are calculated for our device in Supplementary Fig. 9. The negative detuning *Δ* = −21 meV implies that at *θ* = 0 rad emission angle, the photon fraction |*C*_0_|^2^ is much larger than the exciton fraction |*X*_0_|^2^, resulting in a weak interaction of the polariton state with the magnetic field. At *θ* = 0 rad, the Zeeman splitting for the two circular polarization states is $\varDelta {E}_{{{{{{\rm{R}}}}}}-{{{{{\rm{L}}}}}}}={g}_{{{{{{\rm{LP}}}}}}}{\mu }_{{{{{{\rm{B}}}}}}}B$, with ${g}_{{{{{{\rm{LP}}}}}}}=0.41\pm 1.19$ the polariton g-factor and *μ*_B_ the Bohr magneton. This value is not statistically significant since the noise is higher than the energy splitting with magnetic field. At larger emission angles, the Zeeman splitting is higher due to the increase in the exciton fraction of the lower polariton. At *θ* = 0.15 rad, the g-factor becomes *g*_LP_ = 1.06 ± 0.28. The exciton g-factor can be calculated as ${g}_{{{{{{\rm{X}}}}}}}=\frac{{g}_{{{{{{\rm{LP}}}}}}}}{{\left|{X}_{0.15}\right|}^{2}}=3.53\pm 0.93$, in good agreement with previous reports^52^. Exciton polaritons from the K and K’ valleys have the same energy and effective mass at *B* = 0 T, but they preserve the circular polarization associated to the spin-orbit (SO) coupling of the bare monolayer exciton. Supplementary Fig. 11c demonstrates how EL circular polarization can be tuned with external magnetic fields.

### Data analysis

Electroluminescence polarization is defined as ${\rho }_{{{{{{\rm{EL}}}}}}}=({{I}_{{{{{{\rm{K}}}}}}}-{I}_{K{{\hbox{'}}}}})/({{I}_{{{{{{\rm{K}}}}}}}+{I}_{K{{\hbox{'}}}}})-{\rho }_{{EL}}^{0}$, where *I*_K_ (${I}_{{{{{{\rm{K}}}}}}{{\hbox{'}}}}$) corresponds to right (left) circularly polarized intensity and ${\rho }_{{{{{{\rm{EL}}}}}}}^{0}$ corresponds to the circular polarization obtained when *V*_lat_ = 0 and *V*_sq_ = 16 V.

The extracted polarization in Fig. 5 is the weighted arithmetic mean of the polarization of each pixel in the image of the Fourier plane, ${\rho }_{{{{{{\rm{EL}}}}}}}^{{{{{{\rm{w}}}}}}}=\frac{\sum {\rho }_{{{{{{\rm{EL}}}}}}}^{{{{{{\rm{i}}}}}}}{I}_{{{{{{\rm{T}}}}}}}^{{{{{{\rm{i}}}}}}}}{\sum {I}_{{{{{{\rm{T}}}}}}}^{{{{{{\rm{i}}}}}}}}$, where ${I}_{{{{{{\rm{T}}}}}}}^{{{{{{\rm{i}}}}}}}={I}_{{{{{{\rm{K}}}}}}}^{{{{{{\rm{i}}}}}}}+{I}_{{{{{{\rm{K}}}}}}{{\hbox{'}}}}^{{{{{{\rm{i}}}}}}}$ is the total intensity for pixel i. Pixels with intensity *I* < 0.35 *I*_max_ are not taken into account, to reduce noise. Similarly, the emission angle is calculated as ${\theta }_{{{{{{\rm{EL}}}}}}}^{{{{{{\rm{w}}}}}}}=\frac{\sum {\theta }_{{{{{{\rm{EL}}}}}}}^{{{{{{\rm{i}}}}}}}{I}_{{{{{{\rm{T}}}}}}}^{{{{{{\rm{i}}}}}}}}{\sum {I}_{{{{{{\rm{T}}}}}}}^{{{{{{\rm{i}}}}}}}}$. The EL intensity in Fig. 5 corresponds to the total integrated intensity for all pixels in the detector, with the subtraction of the dark counts ${I}_{{{{{{\rm{EL}}}}}}}^{{{{{{\rm{T}}}}}}}=\sum ({I}_{{{{{{\rm{EL}}}}}}}^{{{{{{\rm{i}}}}}}}-{I}_{{{{{{\rm{EL}}}}}}}^{{{{{{\rm{dark}}}}}}})$.

For magnetic-field-dependent EL measurements, the degree of circular polarization ${\rho }_{{{{{{\rm{EL}}}}}}}$ is calculated by taking the average polarization of the *N* = 130 values around the peak energy, corresponding to an energy window of *k*_B_*T*/2, with *k*_B_ the Boltzmann constant and *T* = 300 K. The emission energy *E*_EL_ is calculated as a weighted average over same energy window, with ${E}_{{{{{{\rm{EL}}}}}}}=\frac{\sum {E}^{{{{{{\rm{i}}}}}}}{I}_{{{{{{\rm{T}}}}}}}^{{{{{{\rm{i}}}}}}}}{\sum {I}_{{{{{{\rm{T}}}}}}}^{{{{{{\rm{i}}}}}}}}$, where *E*^i^ is the emission energy for a point in the spectra and ${I}_{{{{{{\rm{T}}}}}}}^{{{{{{\rm{i}}}}}}}={I}_{{{{{{\rm{K}}}}}}}^{{{{{{\rm{i}}}}}}}+{I}_{{{{{{\rm{K}}}}}}{{\hbox{'}}}}^{{{{{{\rm{i}}}}}}}$ the corresponding intensity.

The standard errors are computed considering the error in the intensity of each pixel *I*_K_ as *ΔI*_K_ = 60 cts (intensity fluctuations), the error in the spectral energy *E* as *ΔE* = 0.002 meV (spectrometer resolution) and the error in the measured angle *θ* as *Δθ* = 0.03 rad (angular resolution).

## Supplementary information

Supplementary Information

## Data Availability

The data generated in this study have been deposited in Zenodo database at 10.5281/zenodo.6850668.
