# Supplementary material for: Room-temperature electrical control of polarization and emission angle in a cavity-integrated 2D pulsed LED
Source: Nat Commun. 2022 Aug 19;13:4884. doi: 10.1038/s41467-022-32292-2 (PMC9391484; doi:10.1038/s41467-022-32292-2)
Supplement: Supplementary file 1 — Supplementary Information [file 41467_2022_32292_MOESM1_ESM.pdf]

Supplementary Information

for

Room-temperature electrical control of polarization and emission angle in a cavity-integrated 2D pulsed LED

Juan Francisco Gonzalez Marin[1,2], Dmitrii Unuchek[1,2], Zhe Sun[1,2], Cheol Yeon Cheon[1,2], Fedele Tagarelli[1,2], Kenji Watanabe[3], Takashi Taniguchi[4] & Andras Kis[1,2*]

[1]*Institute of Electrical and Microengineering, École Polytechnique Fédérale de Lausanne (EPFL), CH-1015 Lausanne, Switzerland*
[2]*Institute of Materials Science and Engineering, École Polytechnique Fédérale de Lausanne (EPFL), CH-1015 Lausanne, Switzerland*
[3]*Research Center for Functional Materials, National Institute for Materials Science, 1-1 Namiki, Tsukuba 305-0044, Japan*
[4]*International Center for Materials Nanoarchitectonics, National Institute for Materials Science, 1-1 Namiki, Tsukuba 305-0044, Japan*

**Correspondence should be addressed to: Andras Kis, andras.kis@epfl.ch*

1. Device characterization by AFM

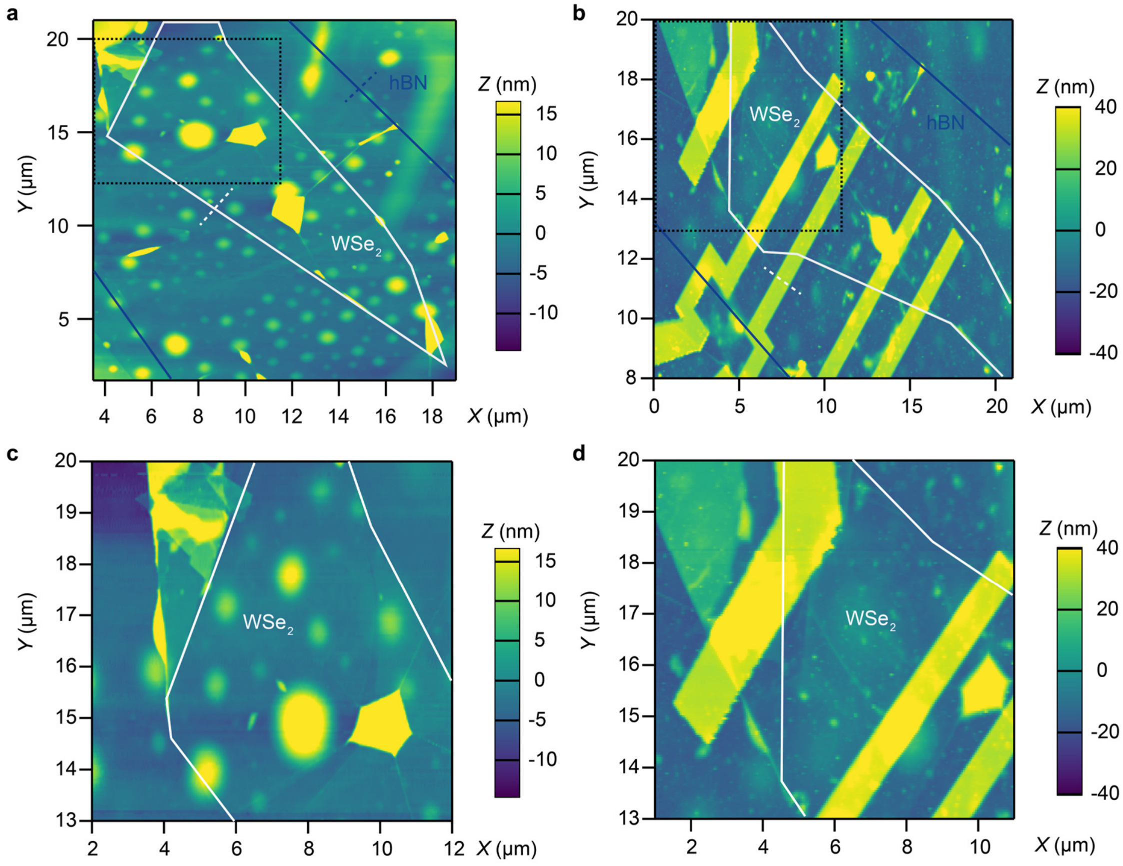

Supplementary Figure 1. AFM characterization. **a,** AFM image after heterostructure fabrication (DBR/Gr/hBN/WSe_2/hBN) and annealing at 340 °C. WSe_2 and top hBN flakes are highlighted with white and blue lines, respectively. **b**, AFM image after contact evaporation and top DBR deposition. White and blue dashed lines are used for the cross sections in Figure S2. **c**, **d,** Zoomed-in AFM images at the EL location for different values of V_{lat} (dashed squares in a, b).

2. Device cross-sections by AFM

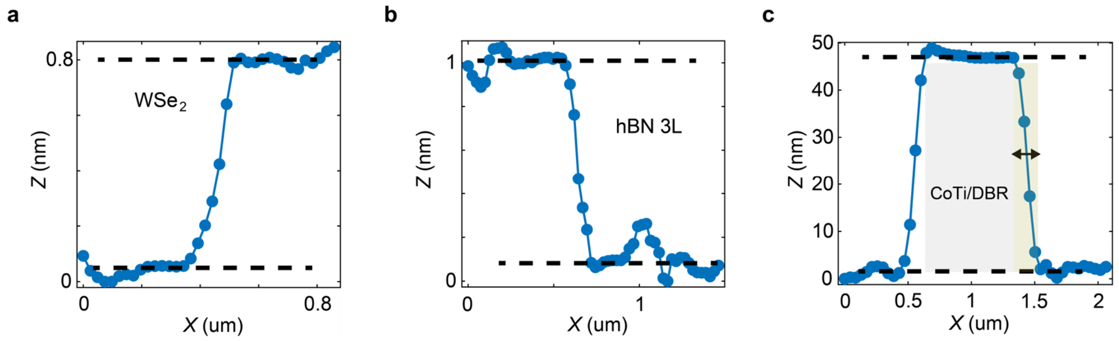

Supplementary Figure 2. AFM characterization**. a, b,** AFM cross section for height identification of WSe_2 and hBN. **c**, AFM cross section on metal contacts after DBR deposition, demonstrating the conformal character of PECVD growth. The yellow rectangle highlights the aspect ratio reached with PECVD growth.

3. Optical characterization of mirrors

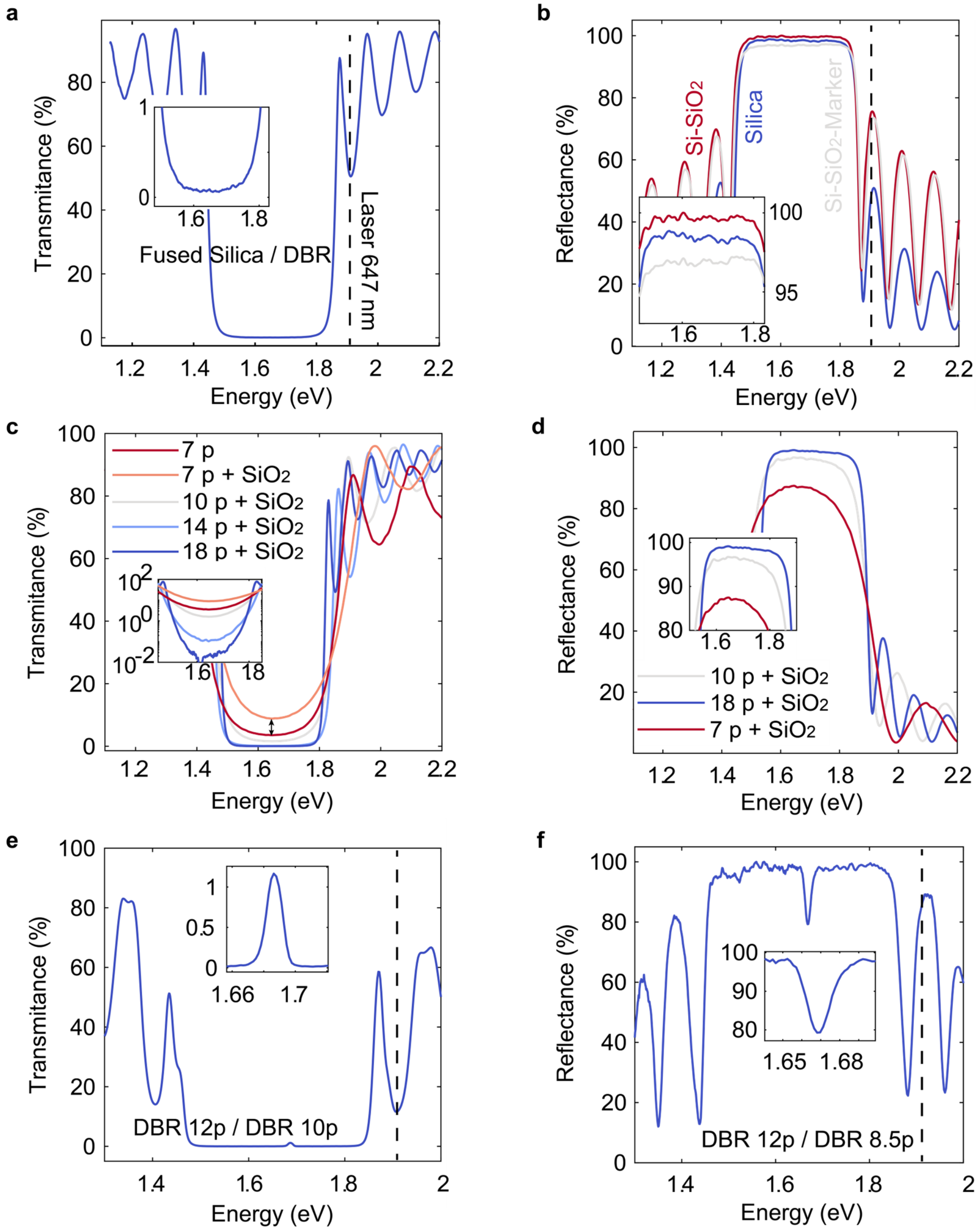

Supplementary Figure 3. Optical characterization of DBR mirrors. **a**, **b**, Transmittance and reflectance for 12 pairs of Ta_2O_5/SiO_2 DBR mirrors grown on different substrates. **c**, **d**, Transmittance and reflectance for SiN/SiO_2 DBR mirrors with different number of dielectric pairs. **e**, **f** Transmittance and reflectance of cavities based on bottom Ta_2O_5/SiO_2 and top SiN/SiO_2 mirrors.

4. Electric field profile

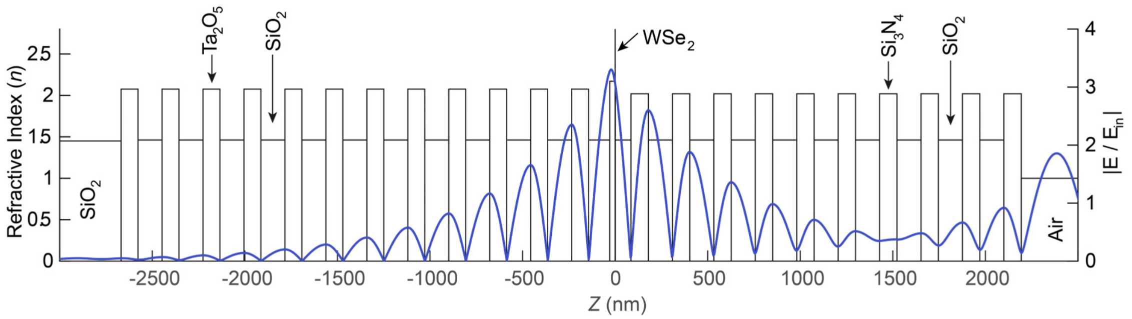

Supplementary Figure 4. Transfer matrix simulations. **a,** Simulated electric field profile for the cavity structure of the device in the main text, considering optical excitation from the top mirror at the resonant wavelength of $\lambda = 761$ nm. Black squares correspond to the refractive index values of the different layers.

5. Gate-voltage-dependent photoluminescence at 5 K

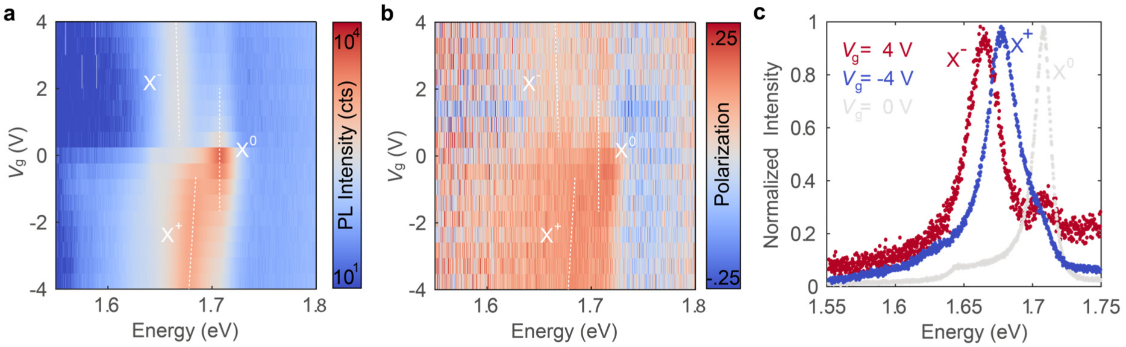

Supplementary Figure 5. Gate dependence of photoluminescence at 5 K. **a,** PL intensity as a function of gate voltage. White dashed lines indicate the position of the different excitonic species. **b**, Extracted PL polarization as a function of gate voltage. **c**, Normalized PL spectra at different carrier densities.

6. Gate-voltage-dependent photoluminescence at 300 K

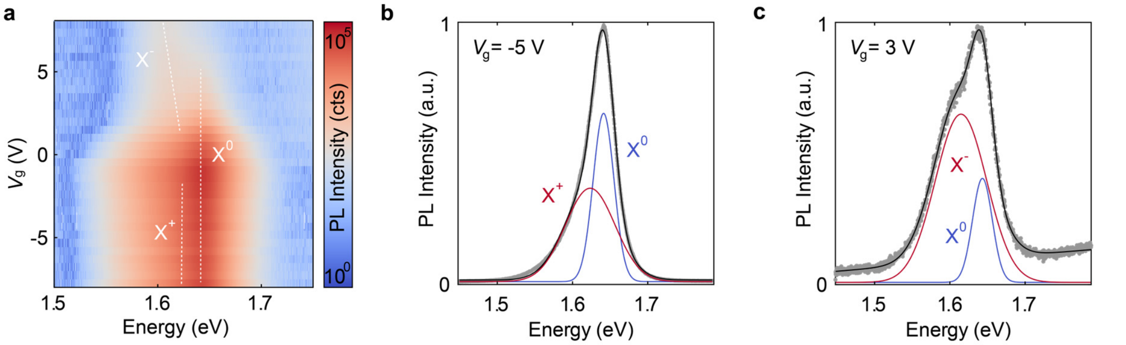

Supplementary Figure 6. Gate dependence of photoluminescence at 300 K. **a,** PL intensity as a function of gate voltage. White dashed lines indicate the position of the different excitonic species. **b**, PL spectra at V_g = -5 V. **c**, PL spectra at V_g = 3 V. Black lines correspond to a Gaussian fit of the data. Red and blue lines correspond to the trion and neutral exciton components extracted from the fit. The neutral exciton energy and linewidths are $E_{\mathrm{X}} = 1.643 \pm 0.001$ meV and $\gamma_{\mathrm{X}} = 27.9 \pm 0.2$ meV. For the positive trion, $E_{\mathrm{X}^+} = 1.624 \pm 0.001$ meV and $\gamma_{\mathrm{X}^+} = 78.7 \pm 0.2$ meV. For the negative trion, $E_{\mathrm{X}^-} = 1.615 \pm 0.001$ meV and $\gamma_{\mathrm{X}^-} = 80.3 \pm 0.1$ meV.

7. Reflectance map after cavity growth

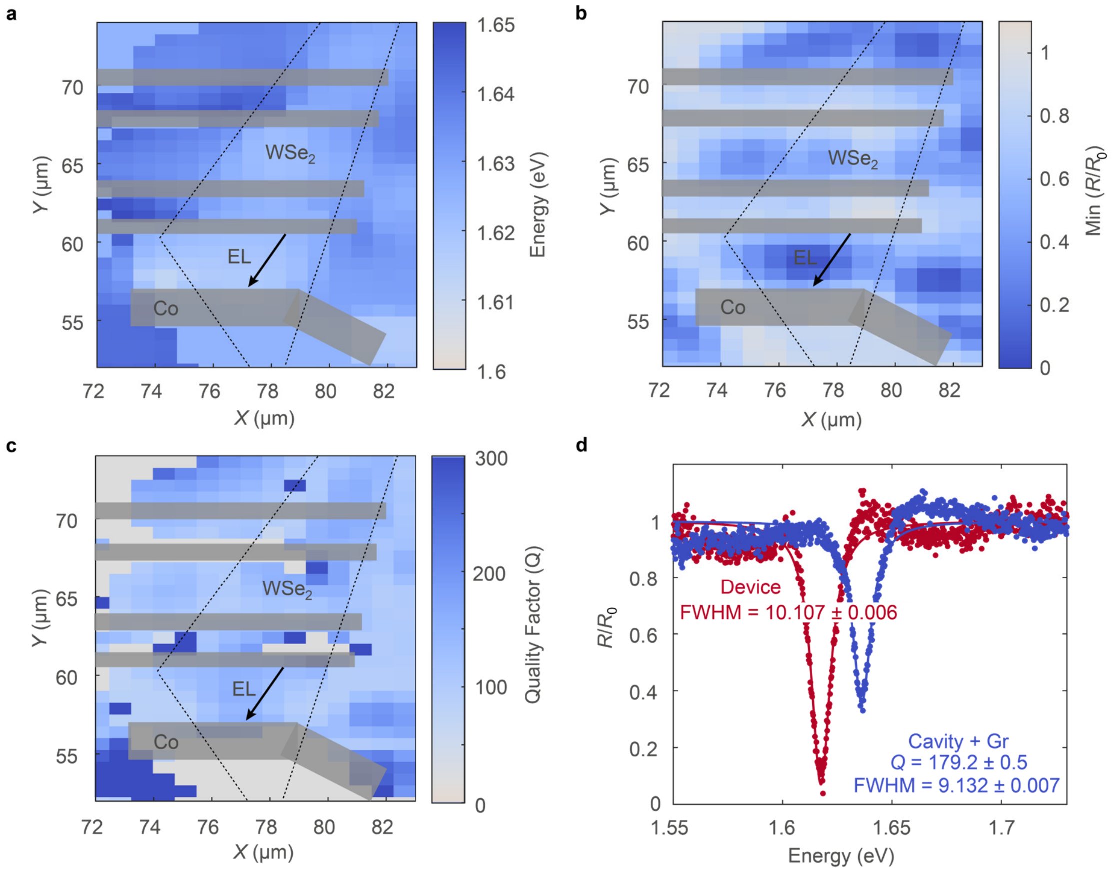

Supplementary Figure 7. Reflectance at $T = 300\,\mathrm{K}$ after top DBR deposition. **a,** Cavity mode energy. **b**, Normalized reflectance at the cavity energy. **c**, Quality factor extracted from Lorentzian fits of reflectance. Black dashed lines outline the monolayer of WSe_2. The arrow indicates the EL location at different values of V_{lat}. The cavity mode energy along the EL path is in the range [1.617,1.622] eV. The quality factor is in the range [102, 133.7]. **d**, Normalized reflectance together with Lorentzian fits for the Gr/hBN/hBN cavity (blue) and Gr/hBN/WSe_2/hBN (red).

8. Reflectance dispersion after cavity growth

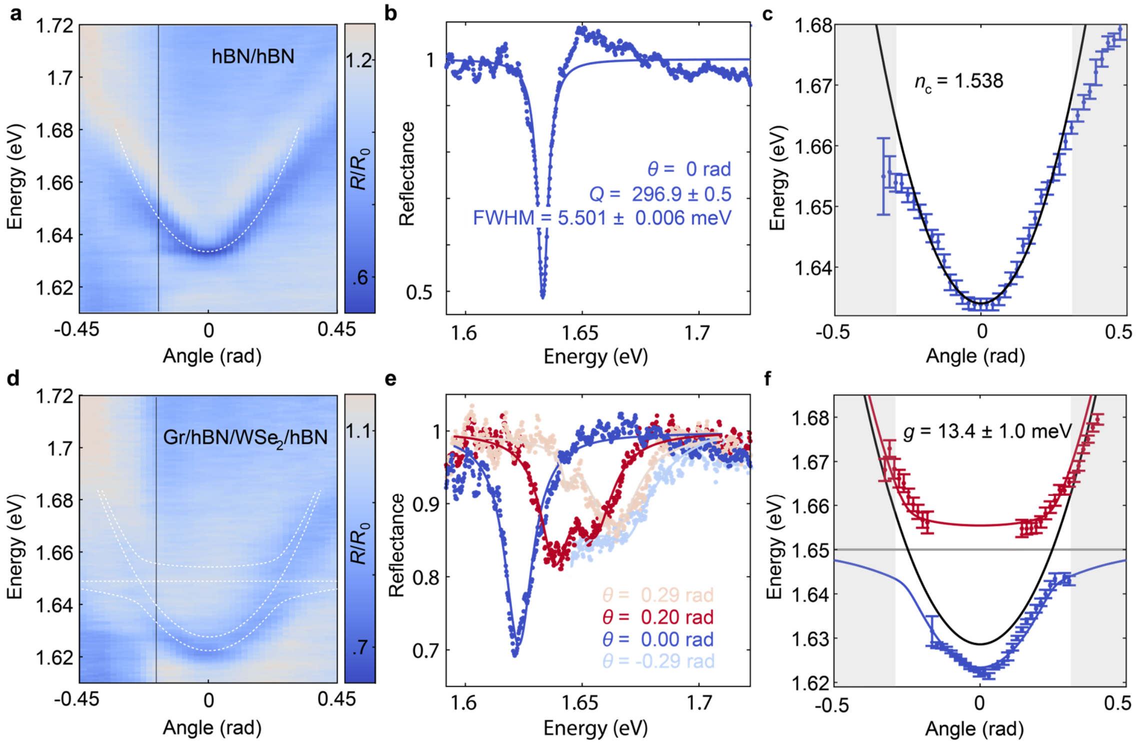

Supplementary Figure 8. Reflectance dispersion at $T = 300\,\mathrm{K}$ after top DBR deposition. **a,** Reflectance dispersion of the bare cavity. The white dashed line indicates the theoretical dispersion. The vertical black line indicates angles below which the signal is comparable to the noise. **b**, Reflectance spectra at $\theta = 0\,\mathrm{rad}$ together with Lorentzian fit to the data. **c**, Calculated cavity mode energy (blue) together with simulated energy dispersion (black) based on the effective refractive index of the cavity. **d**, Reflectance dispersion of the device heterostructure. **e**, Reflectance spectra at different angles together with double Lorentzian fits to the data. **f**, Calculated upper (red) and lower (blue) polariton energies together with the fitted energy dispersion considering a model of two damped oscillators with coupling constant g.

9. Simulations of strong light-matter interaction

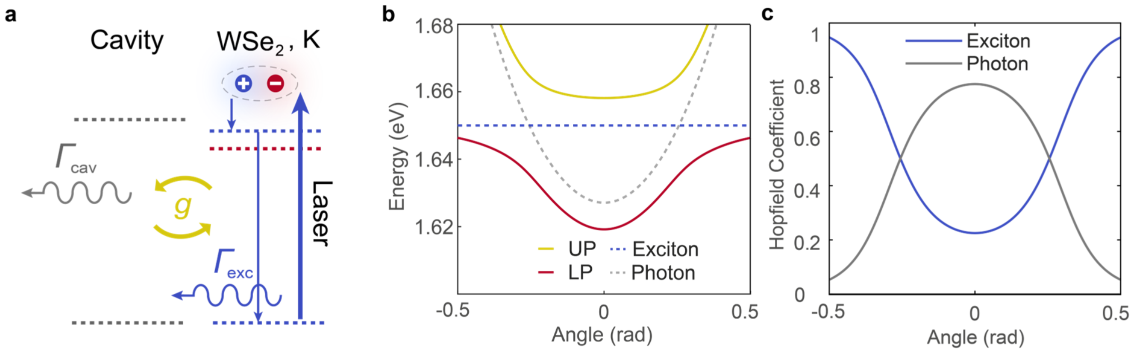

Supplementary Figure 9. Exciton-polariton dispersion and Hopfield coefficients. **a,** Exciton-cavity coupled system including cavity losses and exciton pumping. **b**, Simulated energy dispersion as a function of emission angle in the strong coupling regime for a detuning of $\Delta = -21\,\mathrm{meV}$ and coupling constant of $g = 17.4\,\mathrm{meV}$. **c**, Hopfield coefficients for the simulation in **b**.

10. Electroluminescence emission energy

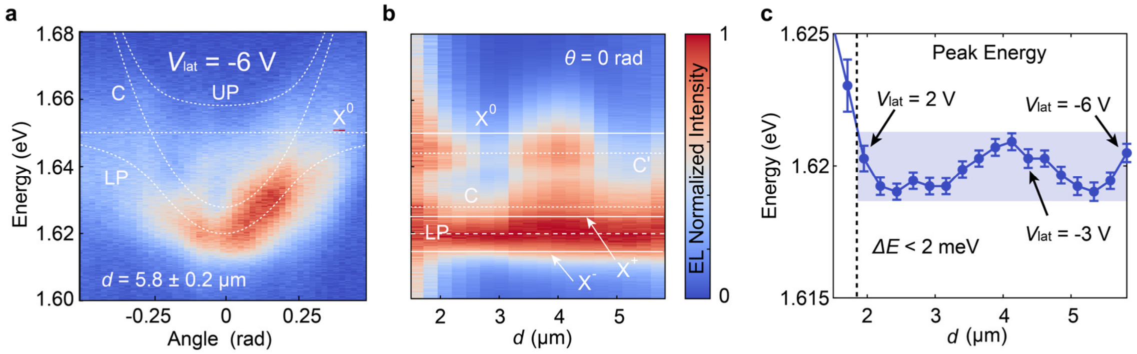

Supplementary Figure 10. Polariton energy along propagation direction. **a,** EL dispersion at $d = 5.8 \pm 0.2\,\mu\mathrm{m}$. **b**, EL spectra at $\theta = 0$ rad as a function of the distance from the source contact to the EL location. The position of charged trions is obtained from Supplementary Figure 6. The position of the second cavity mode C' is obtained from Figure 4b. **c**, Extracted peak emission energy of the lower polariton.

11. Magnetic field dependence of electroluminescence

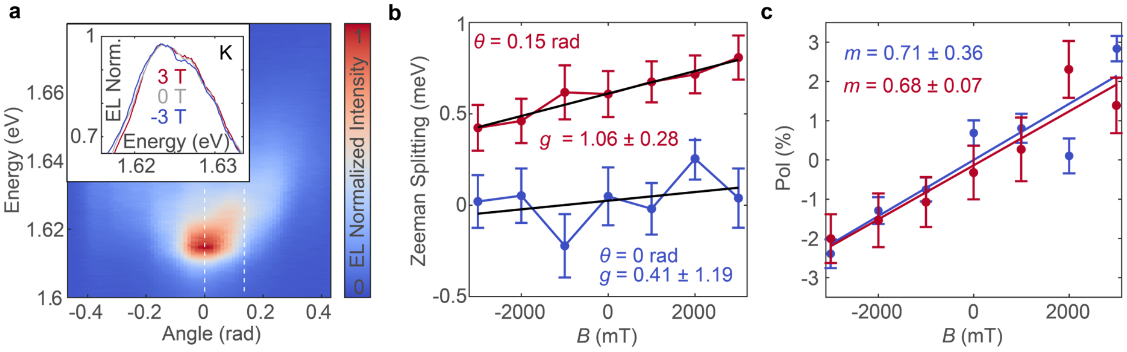

Supplementary Figure 11. Magnetic field dependence of EL at $T = 300$ K. **a,** EL dispersion at $B = 0$ T. White dashed lines indicate the angles taken for the calculation of Zeeman splitting. Inset shows the EL spectra from the K valley at $\theta = 0.15$ rad and at three representative values of the external magnetic field, where the energy shift is visible. **b**, Extracted Zeeman splitting for polaritons at emission angles of $\theta = 0$ rad and $\theta = 0.15$ rad. Black lines are linear fits to the data for the extraction of the g-factor **c**, Magnetic field dependence of EL polarization for $\theta = 0$ rad (blue) and $\theta = 0.15$ rad (red).

12. Simulation of exciton-photon momentum and energy conservation

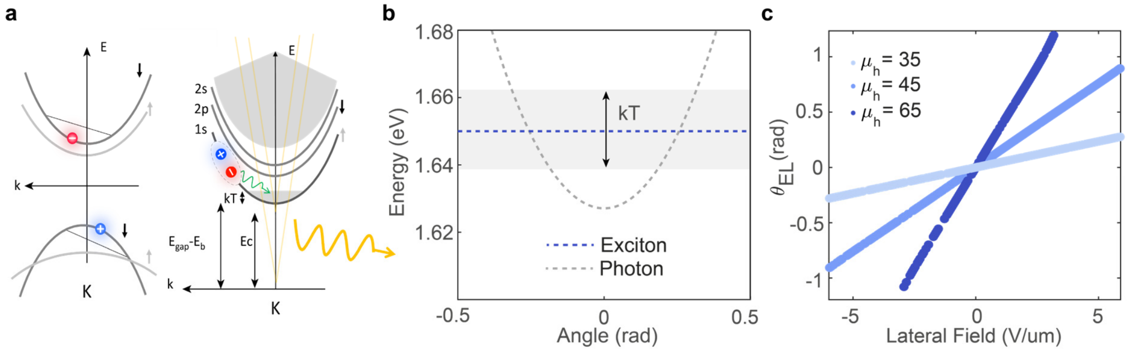

Supplementary Figure 12. Exciton-photon energy and momentum conservation. **a,** Left: schematics representing the tilt in the quasi Fermi levels of electrons and holes in the presence of a lateral electric field. Right: exciton formation with finite momentum k after binding of an electron hole pair. The interplay between initial exciton momentum, relaxation time and radiative lifetime determines the resulting photon emission angle out of the cavity. **b**, Energy dispersion for excitons and cavity photons as a function of the emission angle. **c**, Simulated EL emission angle as a function of the lateral electric field for different values of hole mobilities and fixed electron mobility $\mu_e = 40\ \mathrm{cm}^2\ /\ \mathrm{V\ s}$.

13. Electroluminescence polarization without cavity at T = 4 K

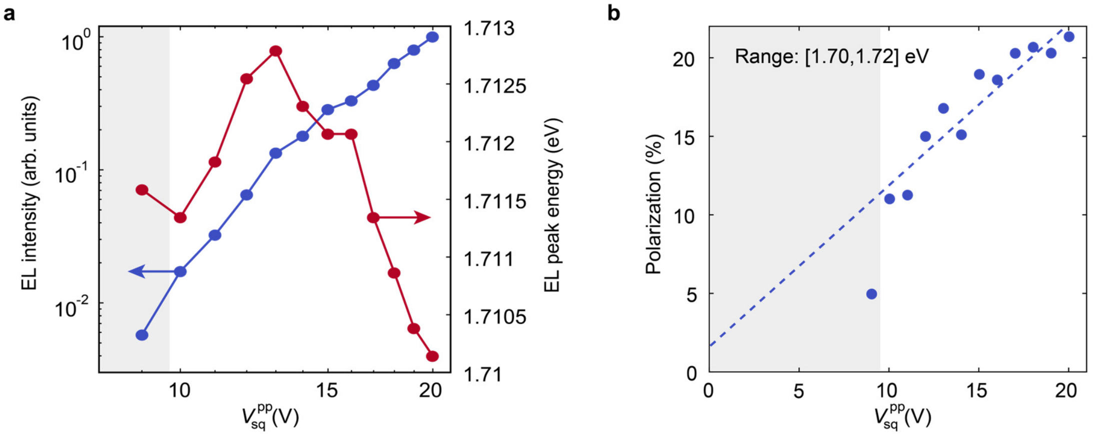

Supplementary Figure 13. EL dependence on the peak-to-peak voltage ($V_{\mathrm{sq}}^{\mathrm{pp}}$) applied to the source electrode. **a**, EL intensity and peak emission energy as a function of the driving voltage amplitude. By comparing the EL energy shift with the one from Figure 2c of the main text, we extract a corresponding temperature change of 40 K due to Joule heating. **b**, Polarization dependence of EL at the neutral exciton energy, with a linear fit excluding $V_{\mathrm{sq}}^{\mathrm{pp}} = 9\ \mathrm{V}$. The gray boxes contain the points with emission intensity below the noise floor.

14. Polarization dependence on the electric field direction at T = 4 K

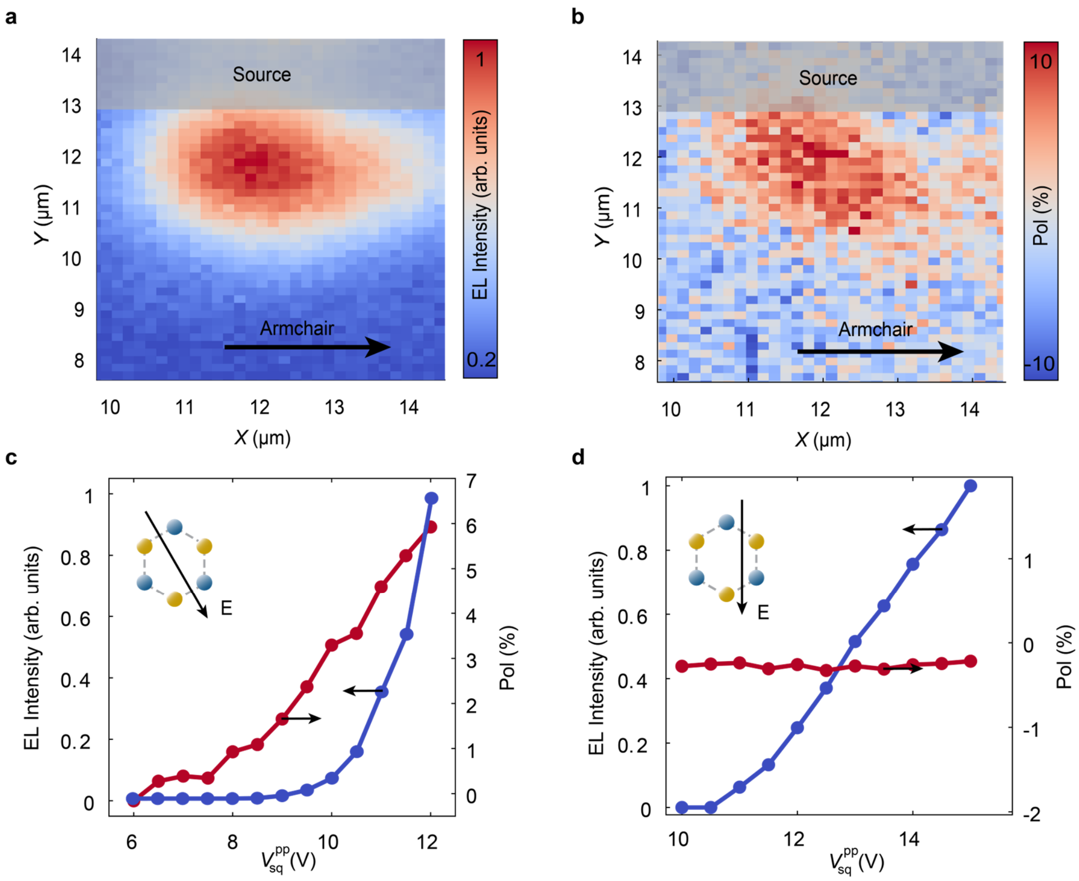

Supplementary Figure 14. Electroluminescence polarization for different contact placements. **a,** Normalized EL intensity for $V_{sq}^{pp} = 10\,\mathrm{V}$ and $f = 6\,\mathrm{MHz}$. The $\mathrm{WSe_2}$ flake covers the whole area and the metal contact is placed along the armchair direction of the monolayer. **b,** Extracted circular polarization. **c**, Dependence of the EL intensity and polarization on the source voltage V_{pp} for the contact drawn in a. **d**, Dependence of the EL intensity and polarization on the source voltage for a contact placed along the zigzag direction, where no polarization is observed even at large applied voltages.

15. Effect of the valence band anisotropy on exciton dispersion

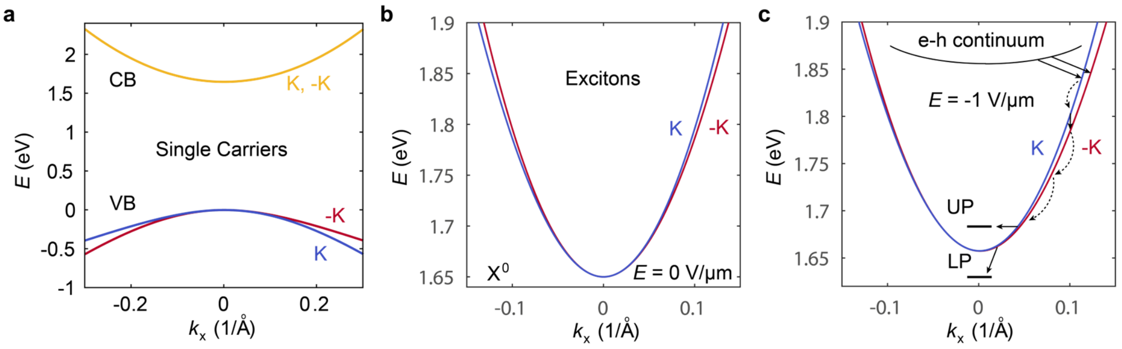

Supplementary Figure 15. Effect of the valence band anisotropy on exciton dispersion. **a,** Energy dispersion for the conduction and the valence bands at the K and K' valleys. **b,** Exciton dispersion at zero field. **c**, Exciton dispersion and relaxation under a lateral electric field. Black dashed arrows correspond to energy relaxation along the exciton states. Black continuous arrows indicate the transition to the exciton and polariton states.

16. Dependence of electroluminescence on the objective position

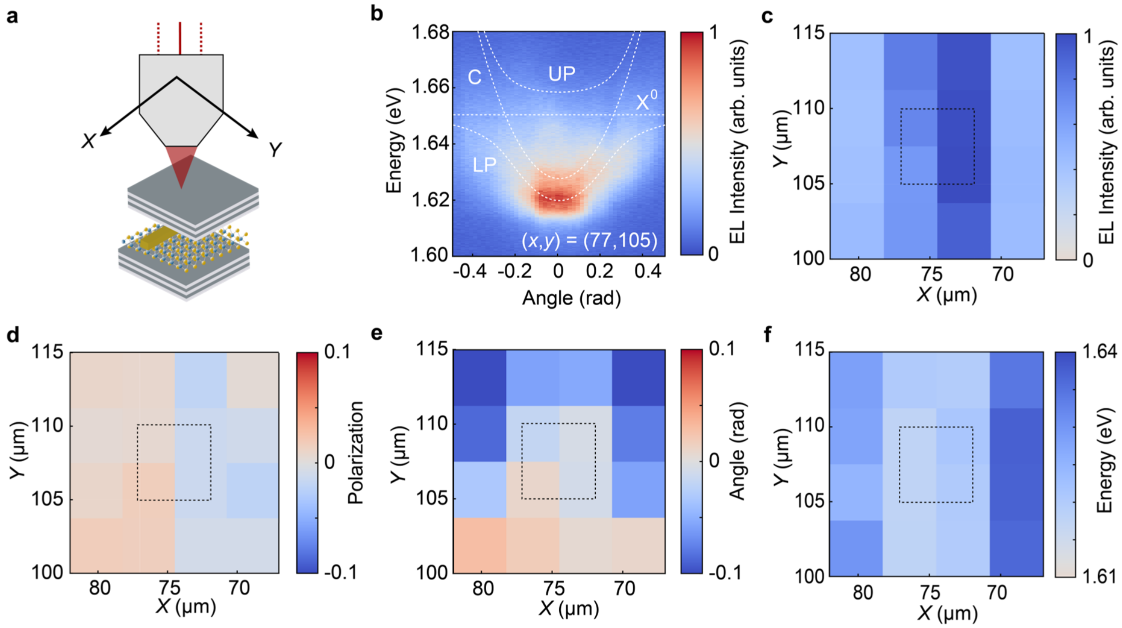

Supplementary Figure 16. Variation of EL dispersion with objective position. **a,** Schematics of the measurement configuration. **b,** EL dispersion for a representative position of the objective. **c**, Normalized integrated intensity of EL. **d**, Weighted average polarization. **e**, Weighted average emission angle. **f**, Peak emission energy. The dashed square represents the maximum EL displacement observed in Figure 3a with respect to the position of the objective. The influence in the resulting polarization ($\Delta P < 3$ %) and angular emission ($\Delta\theta < 0.03$ rad) is negligible.

17. Photoluminescence dispersion map at T = 300 K

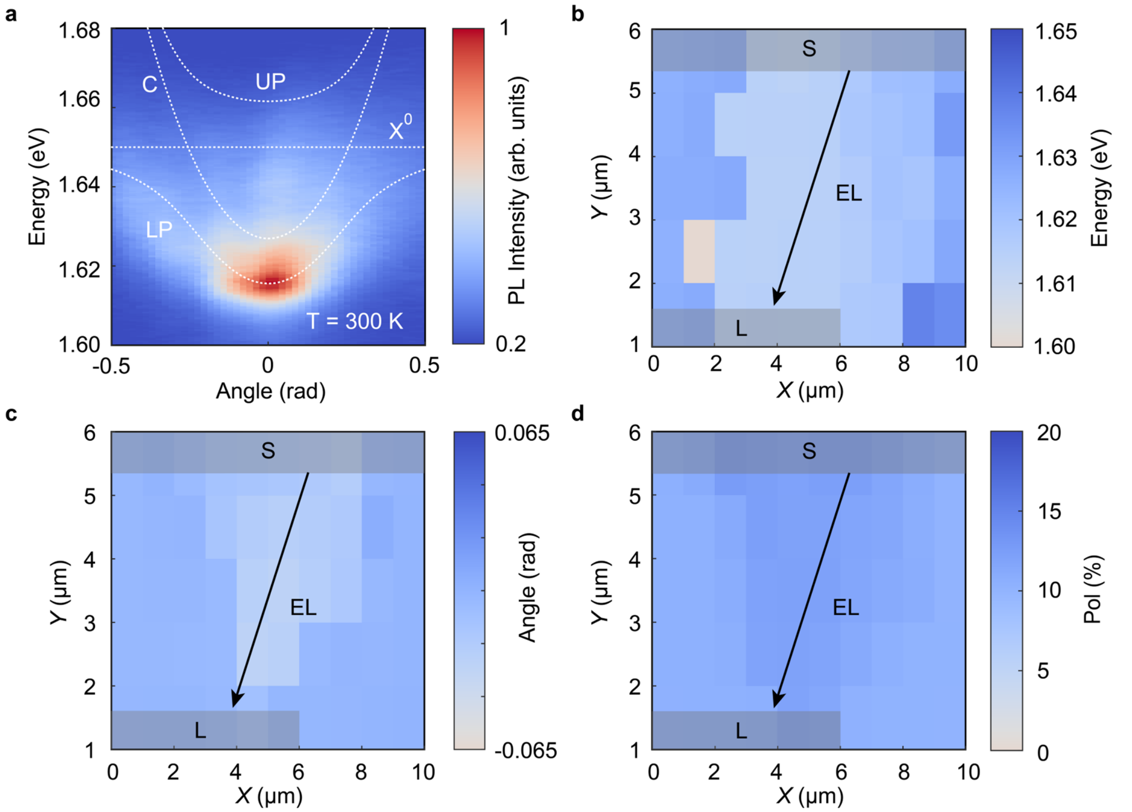

Supplementary Figure 17. PL dispersion map. **a,** Representative PL dispersion at $T = 300\,\mathrm{K}$. **b,** PL peak emission energy. **c**, Weighted average emission angle. **d**, Weighted average polarization. The black arrow represents the EL location at different lateral voltages. The spatial variability of the cavity coupled photoluminescence in terms of polarization (ΔP < 4 %) and angular emission ($\Delta\theta$ < 0.04 rad) is negligible with respect to the electrical modulation demonstrated in the main text.
